# Autologous stem cell transplantation in adult patients with intermediate-risk acute myeloid leukemia in first complete remission and no detectable minimal residual disease. A comparative retrospective study with haploidentical transplants of the global committee and the ALWP of the EBMT

**DOI:** 10.1038/s41409-023-02070-9

**Published:** 2023-08-28

**Authors:** Jia Chen, Myriam Labopin, Thomas Pabst, Xi Zhang, Erlie Jiang, Alessandra Tucci, Jan Cornelissen, Ellen Meijer, Irma Khevelidze, Emmanuelle Polge, Depei Wu, Mohamad Mohty, Norbert-Claude Gorin

**Affiliations:** 1 National Clinical Research Center for Hematologic Diseases, Jiangsu Institute of Hematology, The First Affiliated Hospital of Soochow University, Suzhou, China; 2 Department of Clinical Hematology and Cellular Therapy, Saint-Antoine Hospital, AP-HP, Sorbonne University, EBMT Paris office, Paris, France; 3 Department of Medical Oncology, Inselspital, Bern University Hospital, Bern, Switzerland; 4 Medical center of hematology, Xinqiao Hospital, Army Medical University, Chongqing, China; 5 Institute of Hematology, Chinese Academy of Medical Sciences, Hematopoietic stem cell transplantation center, Tianjin, China; 6 Spedali Civili – Brescia, Hematology Division, Department of Medical Oncology, Brescia, Italy; 7 Erasmus MC Cancer Institute, University Medical Center Rotterdam, Department of Hematology, Rotterdam, Netherlands; 8 VU University Medical Center, Department of Hematology, Amsterdam, Netherlands

**Keywords:** Diseases, Acute myeloid leukaemia

## Abstract

In patients with acute myeloid leukemia (AML) of intermediate-risk (IR) in first remission (CR1) with no measurable residual disease (MRD negative), the choice of the best consolidation is questionable. 1122 adult patients from 196 centers, transplanted in 2010-21 were analyzed: 547 received an autologous stem cell transplantation (ASCT) and 575 a Haploidentical donor transplant. Because of a significant interaction, comparisons were done separately for patients with wild-type FLT3 (FLT3-wt) and FLT3-ITD mutation (FLT3-ITD). In FLT3-wt patients, haploidentical transplants had two year lower relapse incidence (RI) (16.9% versus 32.6%; HR = 0.40, *p* < 0.001), higher NRM higher (17.2% vs 3.5%; HR = 7.02, *p* < 0.001), similar LFS (65.9% vs 63.8%; *p* = 0.37) and lower OS (73.2% vs 80.6%; HR = 1.69, *p* = 0.018). In FLT3-ITD patients, haploidentical transplants had two year lower RI (8.2% vs 47.8%; HR = 0.14, p < 0.001) higher NRM (20.2% vs 5.6%; HR = 3.43, *p* = 0.002), better LFS (71.5% vs 46.6%; HR = 0.53, *p* = 0.007) and similar OS (73.5% vs 61.9%; *p* = 0.44). In IR AML patients with FLT3-wt in MRD negative CR1, autologous stem cell transplantation is a valid option, while in patients with FLT3-ITD, haploidentical transplant is better. Whether autologous transplantation is superior to chemotherapy in FLT3-wt patients and the role of maintenance therapy with FLT3 inhibitors remain to be studied.

## Introduction

In the past decade there have been several major improvements in the field of acute myeloid leukemia (AML). These have included the identification of several new molecular markers leading to better monitoring of the disease and development of several new targeted therapies [1]. Recent data have shown the importance of reaching a status of undetectable measurable residual disease (MRD negative) at all steps of the therapeutic pathway and has led to redefining the notion of MRD negative complete remission (CR) [2–4]. In Europe, allogeneic hematopoietic stem cell transplantation (HSCT) has been considerably boosted with the introduction of T cell replete haploidentical donor HSCT using mostly reduced intensity conditioning and high dose cyclophosphamide post-transplant (PTCY) to prevent graft-versus-host disease (GVHD), following the pioneering work from the Johns Hopkins group in Baltimore [5–7]. The situation has been similar in China with the protocol developed by the Beijing team [8–11], which uses myeloablative conditioning and primed bone marrow (BM) with mobilized peripheral blood stem cells (PBSC) as the stem cell source. With the availability of either matched siblings or unrelated donors and now haploidentical donor HSCT, most patients with AML in need of a transplant have access to one or more potential donors and the risk of grade II-IV acute or severe chronic GVHD, although still present, has been considerably reduced. The development of several new therapeutic agents including combinations such as venetoclax and azacytidine [12, 13], and new targeted therapies, mainly inhibitors of FLT3 mutations [14–22] has generated clinical trials of maintenance therapy to reduce the risk of relapse post-allogeneic HSCT.

As it stands today and following consensus guidelines, fit patients with AML with adverse risk factors and/or failure to achieve MRD negative CR as well as patients in relapse are all offered an allogeneic HSCT as the only potential option leading to cure. In contrast, in the absence of randomized studies, the nature of the consolidation therapy for favorable and intermediate-risk (IR) patients in first MRDneg first CR (IR-CR1-MRD negative) remains controversial.

Autologous stem cell transplantation (ASCT) which has been widely used before the year 2000 to consolidate patients in CR1, has become out of favor mainly because of a higher relapse incidence (RI) despite a reduced non relapse mortality (NRM) [23]. However, several studies have shown improvement of outcome post ASCT in patients in MRD negative remission. A recent European Society for Blood and Marrow Transplantation (EBMT) study in patients in first molecular remission comparing ASCT to allogeneic transplants with 10/10 unrelated donor transplants has shown superior outcome with ASCT in favorable-risk patients as well as identical outcome in intermediate-2 risk patients, defined by the 2010 European Leukemia Net (ELN) classification [24], with, in addition, a likely better quality of life.

The nature of the transplant to apply to patients in the intermediate-risk categories especially if in CR1 MRD negative remains a matter of debate and is said to correspond to a gray decision zone. There are situations where the only available donor for an allogeneic transplant is a haploidentical donor: for instance in China, because of the one child policy in application until recently, the only possible choice for long has been whether to select a haploidentical donor or do a myeloablative consolidation with an ASCT. This situation also occurs, although less frequently, elsewhere on the globe.

Here we compared the outcome postASCT or haploidentical transplants in patients with intermediate-risk AML achieving CR1 and MRD negativity at pretransplant in the period from 2010 to 2021.

## Methods

### Patients and data collection

This study is a retrospective, multicenter analysis. Data were provided by the Acute Leukemia Working Party (ALWP) of the EBMT registry. The EBMT registry is a voluntary working society which regroups more than 550 transplant centers that are required to report all consecutive stem cell transplants and follow-up on an annual basis. Audits are routinely performed to ensure the accuracy of data. Since 1990, registry patients have provided informed consent authorizing the use of their personal information for research purposes. The Global Committee and ALWP of the EBMT approved this study.

The study included all adult patients in the period from January 2010 to January 2021 who received an autograft or a T cell replete haploidentical donor HSCT for consolidation of an intermediate-risk AML (defined by cytogenetics and molecular biology for FLT3 and NPM1) in MRD negative CR1. The following data were collected: patient gender and age at transplant, Karnofsky performance score at transplant and at follow-up, FLT3 and NPM1 molecular markers, disease status, time from diagnosis to CR1 achievement, time from diagnosis to HSCT, details of pretransplant therapy, source of stem cells, patient and haploidentical donor CMV serostatus, relapse and major complications post-transplant, date and cause of death. Subsequent transplants post-relapse and their outcomes were recorded.

The status of minimal residual disease (negative or positive) was defined locally by each team for each individual patient in relation to the presence of a specific molecular marker and/or by flow cytometry and according to the quantification method and detection thresholds in use at each local institution.

### Endpoints and statistical analysis

Leukemia free survival (LFS), defined as the time interval between transplant and relapse or death, was the primary study endpoint. Secondary endpoints were: (i) NRM defined as death without previous relapse; (ii) Relapse incidence (RI) defined on the basis of morphological evidence of leukemia in BM or other extramedullary organs; and (iii) overall survival (OS) defined as the time interval between transplant and death from any cause.

Patients’ characteristics were compared using the Mann-Whitney test for continuous variables, and the chi-squared or Fisher’s exact test for categorical variables. Cumulative incidence curves were used for RI and NRM in a competing risk setting [25], since death and relapse are competing. Probabilities of OS and LFS were calculated using the Kaplan–Meier method. Multivariate analysis was performed using a Cox proportional-hazards model [26] which included variables differing significantly (*p* < 0.05) between the groups, factors known to be associated with outcomes, plus a center frailty effect to take into account the heterogeneity across centers. Missing values were excluded from the multivariate analysis. The follow-up time was calculated using the reverse Kaplan–Meier method. All tests were two-sided with the type I error rate fixed at 0.05.

Statistical analyses were performed with SPSS 27.0 (SPSS Inc., Chicago, IL, USA) and R 4.1.1 (R Development Core Team, Vienna, Austria, URL: https://www.R-project.org/).

## Results

### Patient populations

In the period considered for this study, 3403 adult patients with AML classified as intermediate-risk by cytogenetics, were transplanted in CR1 (2080 haploidentical donor HSCT and 1323 ASCT) and reported to the EBMT registry. Of these, 1426 were MRD negative at time of transplant and 1131 had information on their FLT3 molecular status (386 FLT3-ITD and 745 wild-type). Nine patients lacked follow-up data and were excluded. A total of 1122 patients from 196 centers world-wide were included in the study. The median follow-up was 37.5 months (IQR: 36–40 months). Table 1 shows the distribution of the characteristics of the patients and the transplants.

**Table 1:** Distribution and characteristics of intermediate-risk AML patients transplanted in CR1 MRD negative.

		AUTO group	HAPLO group	p-value
Number of patients		547	575	
Year of transplant	Median (min-max)	2016 (2010–2021)	2018 (2010–2021)	<0.0001
Follow-up (months)	Median [IQR] range	45 [41–49] 1–144	34 [30–36] 1–128	<0.0001
Country	China	63 (11.5%)	164 (28.5)	<0.0001
	Other	484 (88.5%)	411 (71.5)	
Patient age at HSCT (years)	Median [IQR]	54 [43–62]	49 [37–60]	<0.0001
Patient sex	Male	278 (50.8%)	328 (57%)	0.72
	Female	269 (49.2%)	247 (43%)	
Year of HSCT	Median [range]	2016 [2010–2021]	2018 [2010–2021]	<0.0001
FLT3 mutation status	FLT3-wt	418 (76.4%)	325 (56.5%)	<0.0001
	FLT3-ITD	129 (23.6%)	250 (43.5%)	
NPM1 mutation status	NPM1wt	210 (38.4%)	327 (56.9%)	<0.0001
	NPM1 mutated	324 (59.2%)	188 (32.7%)	
	Missing	13 (2.4%)	60 (10.4%)	
Karnofsky score at transplant	<90	82 (15%)	188 (32.7%)	<0.0001
	≥90	433 (79.2%)	335 (58.3%)	
	Missing	32 (5.8%)	52 (9%)	
Patient CMV status	Positive	110 (20.1%)	343 (59.7%)	0.8
	Negative	64 (11.7%)	209 (36.3%)	
	Missing	373 (68.2%)	23 (4%)	
Stem cell source	BM	12 (2.2%)	118 (20.5%)	
	PB	530 (96.9%)	314 (54.6%)	
	BM + PB	5 (0.9%)	45 (7.8%)	
	CB with BM/PB	0	98 (17.1%)	
Conditioning regimen	BU/CY-based	294 (53.8%)	143 (24.9%)	
	BU/MEL-based	61 (11.2%)	0	
	BU/FLU-based	30 (5.5%)	98 (17.1%)	
	FLU/TBI-based	0	55 (9.6%)	
	BUVP16:	45 (8.2%)	0	
	TBF-based	0	203 (35.4%)	
GVHD prevention Associated IS	PTCY	-	292 (51.5%)	
	In vivo TCD	-	184 (32.5%)	
	Both		52 (9.2%)	
	Other		39 (6.8%)	
	CSA + MMF	-	213 (37.6%)	
	CSA + MTX + MMF	-	132 (23.3%)	
	MMF + TACRO	-	115 (20.3%)	
	OTHER	-	107 (18.8%)	
Interval Diag-HSCT (months)	Median [range]	4.7 [1–53]	5.2 [1–61]	0.0003

*IQR* interquartile range, *HSCT* hematopoieticstem cell transplantation, *wt* wild-type, *GVHD* graft-versus-hostdisease, *CMV* cytomegalovirus, *BM* bonemarrow, *PB* peripheralblood, *CB* cordblood, *BU* busulfan, *CY* cyclophosphamide, *MEL* melphalan, *FLU* fludarabine, *TBI* totalbody irradiation, *TBF* thiotepa-busulfan-fludarabine, *PTCy* post-transplantcyclophosphamide, *ATG* anti-thymocyteglobulin, *CSA* cyclosporinA, *MMF* mycophenolatemofetil, *MTX* methotrexate.

Patients autografted were significantly older than those receiving a haploidentical donor HSCT (median age of 54 years vs 49 years, *p* < 0.0001). The incidence of FLT3-ITD was significantly lower in patients autografted (23.6% vs 43.5%, *p* < 0.0001), and their interval from diagnosis to transplant was shorter (4.7 months vs 5.2 months, *p* = 0.0003).

Of note, the pretransplant regimen varied, with the majority of ASCT receiving a combination of Busulfan and Cyclophosphamide (BUCY: 53.8%) or Melphalan (BUMEL: 11.2%) or VP16 (BUVP16: 8.2%) and the majority of haploidentical transplants receiving a combination of Thiotepa, Busulfan and Fludarabin (TBF: 35.4%), BUCY (24.9%) or Busulfan and Fludarabin (FB: 17.1%).

Regarding MRD evaluation, details on the methods used were available in 393 patients. There was no difference between the methods used for ASCT and haploidentical transplants and no difference between the methods used in the FLT3-wt and FLT3-ITD populations (supplementary table 1).

### Transplant outcomes

Failure to engraft was observed in 5 (1%) patients receiving an autograft and 21 (3.7%) receiving a haploidentical donor HSCT (results not shown). As there was a significant interaction between the FLT3-ITD status and the type of transplant on LFS, outcome comparisons were performed separately for patients with wild-type FLT3 (FLT3-wt) and those bearing the FLT-ITD mutation (FLT3-ITD).

#### 1-Patients with FLT3-wt

**Fig. 1:**
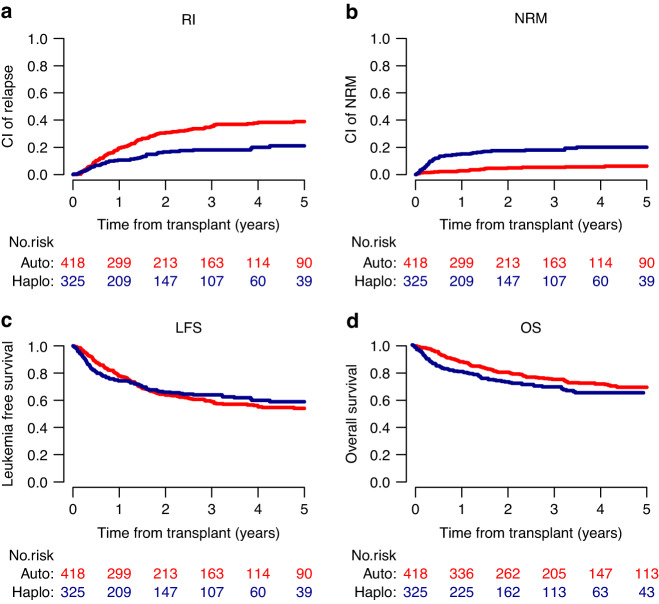
Transplant outcomes of intermediate-risk AML adults with FLT3-wt in CR1 and uMRD: comparison between ASCT group and HAPLO group. **a** Relapse incidence (RI) **b** Non Relapse Mortality (NRM) **c** Leukemia Free Survival (LFS) **d** Overall Survival (OS) comparison between ASCT group and HAPLO group.

As shown in Fig. 1, the haploidentical donor group had a lower 2-year RI (16.9% vs 32.6%, *p* < 0.001), but a higher 2-year NRM (17.2% vs 3.5%, *p* < 0.001) when compared to the ASCT group. Although 2-year LFS was not statistically different between the haploidentical donor HSCT and the ASCT groups (65.9% vs 63.8%, *p* = 0.72) (HR 0.86; 95% CI: 0.62-1.2; *p* = 0.37), 2-year OS was significantly inferior in the haploidentical donor HSCT group than in the ASCT group (73.2% vs 80.6%, *p* = 0.020).

Multivariate analysis showed that in FLT3-wt patients, haploidentical donor HSCT was independently associated with lower RI (HR 0.4; 95% CI: 0.27–60; *p* < 0.0001), higher NRM (HR 7.02; 95% CI: 3.26–15.1; *p* < 0.0001), and lower OS (HR 1.69; 95% CI: 1.09–2.61; *p* = 0.018). Of note, transplant type had no significant impact on LFS (HR 0.86; 95% CI: 0.62–1.2; *p* = 0.37). Other favorable prognostic factors for OS and LFS in FLT3-wt patients were younger age, which was correlated with a lower NRM, and the presence of the NPM1 mutation which was correlated with lower RI. There was no difference in the results between China and other countries (Table 2).

**Table 2:** Multivariate analysis of transplant outcomes in intermediate-risk AML adults with FLT3-wt in CR1 MRD negative.

	RI		NRM		LFS		OS	
	HR (95% CI)	*p*	HR (95% CI)	*p*	HR (95% CI)	*p*	HR (95% CI)	*p*
HAPLO vs ASCT	0.4 (0.27–0.6)	<0.0001	7.02 (3.26–15.08)	<0.0001	0.86 (0.62–1.2)	0.37	1.69 (1.09–2.61)	0.018
Age (per 10 years)	1.12 (0.99–1.26)	0.067	1.89 (1.43–2.49)	<0.0001	1.25 (1.12–1.39)	<0.0001	1.61 (1.38–1.87)	<0.0001
Year of HSCT	1 (0.95–1.06)	0.94	0.93 (0.84–1.03)	0.15	0.99 (0.95–1.04)	0.69	0.94 (0.88–1)	0.04
Female vs male	1.02 (0.76–1.37)	0.89	1.18 (0.69–1.99)	0.55	1.05 (0.81–1.36)	0.71	1.38 (1–1.9)	0.052
Time diagnosis to HSCT (mo)	0.99 (0.95–1.04)	0.79	1.01 (0.96–1.06)	0.64	1.01 (0.98–1.04)	0.59	1.01 (0.98–1.05)	0.51
NPM1 mut vs wt	0.43 (0.3–0.6)	<0.0001	0.94 (0.48–1.82)	0.85	0.5 (0.37–0.68)	<0.0001	0.44 (0.29–0.66)	<0.0001
China vs other countries	0.55 (0.31–0.98)	0.043	1.48 (0.4–5.52)	0.56	0.63 (0.36–1.1)	0.1	0.89 (0.34–2.3)	0.8

*HR* hazard ratio, *CI* confidential interval, *HAPLO* haploidentical stem cell transplantation, *ASCT* autologous stem cell transplantation, *HSCT* hematopoietic stem cell transplantation, *mo* months, *mut* mutation, *wt* wild-type.

#### 2-Patients with FLT3-ITD

**Fig. 2:**
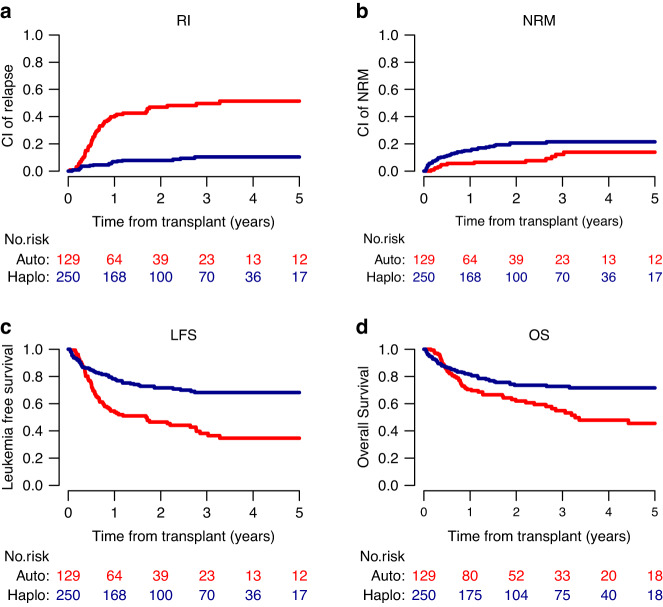
Transplant outcomes of intermediate-risk AML adults with FLT3-ITD in CR1 and MRD neg: comparison between ASCT group and HAPLO group. **a** Relapse incidence (RI) **b** Non Relapse Mortality (NRM) **c** Leukemia Free Survival (LFS) **d** Overall Survival (OS) Autologous stem cell transplantation Haploidentical transplantation.

Similarly, in comparison with ASCT, haploidentical donor HSCT resulted in a lower 2-year RI (8.2% vs 47.8%, *p* < 0.001) and a higher 2-year NRM (20.2% vs 5.6%, *p* = 0.015) in FLT3-ITD patients (Fig. 2). However, the 2-year LFS was markedly superior to that of the ASCT group (71.5% vs 46.6%, *p* < 0.001) as well as the 2-year OS (73.5% vs 61.9%, *p* = 0.012). In addition, more ASCT patients received a subsequent allogeneic HSCT than did the haploidentical donor recipients (20% vs 2.5%, *p* < 0.001).

Results of multivariate analysis of FLT3-ITD patients are displayed in Table 3. In accordance with FLT3-wt patients, haploidentical donor HSCT versus ASCT was significantly associated with a lower RI (HR 0.14; 95% CI: 0.07–28; *p* < 0.0001), and a higher NRM (HR 3.43; 95% CI: 1.55–7.61; *p* = 0.002). In spite of a better LFS (HR 0.53; 95% CI: 0.34–85; *p* = 0.007), transplant type had no statistically significant impact on OS (HR 0.83; 95% CI: 0.52–1.33; *p* = 0.44). Patient younger age and more recent year of HSCT were associated with a lower NRM but this had limited impact on LFS and OS. The presence of the NPM1 mutation remarkably reduced the risk of RI, which translated into superior LFS and OS. There was no difference in the results between China and other countries.

**Table 3:** Multivariate analysis of transplant outcomes in intermediate-risk AML adults with FLT3-ITD transplanted in CR1 MRD negative.

	RI		NRM		LFS		OS	
	HR (95% CI)	*p*	HR (95% CI)	*p*	HR (95% CI)	*p*	HR (95% CI)	*p*
HAPLO vs ASCT	0.14 (0.07–0.28)	<0.0001	3.43 (1.55–7.61)	0.002	0.53 (0.34–0.85)	0.007	0.83 (0.52–1.33)	0.44
Age (per 10 years)	0.86 (0.71–1.05)	0.14	1.54 (1.17–2.02)	0.002	1.09 (0.94–1.27)	0.27	1.19 (1–1.4)	0.048
Year of HSCT	0.95 (0.88–1.03)	0.25	0.87 (0.78–0.97)	0.013	0.93 (0.87–1)	0.04	0.9 (0.84–0.97)	0.004
Female vs male	1.21 (0.73–2)	0.45	1.54 (0.86–2.78)	0.15	1.34 (0.92–1.96)	0.13	1.55 (1.02–2.37)	0.042
Time diagnosis to HSCT (mo)	0.98 (0.88–1.1)	0.76	0.94 (0.81–1.08)	0.36	0.96 (0.88–1.05)	0.38	0.96 (0.88–1.05)	0.37
NPM1 mut vs wt	0.46 (0.26–0.8)	0.006	0.6 (0.33–1.1)	0.1	0.5 (0.33–0.76)	0.001	0.56 (0.36–0.87)	0.009
China vs other countries	1.18 (0.32–4.36)	0.81	0.7 (0.23–2.15)	0.54	0.78 (0.31–1.97)	0.6	0.63 (0.27–1.47)	0.29

*HR* hazard ratio, *CI* confidential interval, *HAPLO* haploidentical stem cell transplantation, *ASCT* autologous stem cell transplantation, *HSCT* hematopoietic stem cell transplantation, *mo* months, *mut* mutation, *wt* wild-type.

## Discussion

Our study addresses the important question of the best transplant consolidation therapy to apply to patients with AML who are in deep CR as attested by the absence of detectable measurable disease and who belong to the intermediate-risk category. As opposed to good risk patients for whom transplantation in CR1 is not recommended, most guidelines recommend consolidation with allogeneic stem cell transplantation for all non-favorable-risk patients either in CR1 or after relapse [1, 27, 28], but in contrast there is presently no consensus about the best consolidation to use in this IR-CR1-MRD negative population of patients studied here. There has been in the past, before MRD evaluation was possible with modern tools, several studies and meta-analysis comparing in younger patients (below 55 years of age) in CR1 by cytology only, chemotherapy alone with ASCT to allogeneic transplantation with matched sibling donors but no study in IR-CR1-MRD negative patients and no study involving transplantation with haploidentical donors.

Regarding the role of further chemotherapy alone, The EBMT registry contains no information on non transplanted patients and our study was not designed to evaluate it. However a considerable number of randomized studies in the past have clearly shown that chemotherapy consolidation is associated with a significantly higher relapse incidence than autologous transplants, which is not surprising since the myeloablative treatment delivered pre ASCT is designed to combine the maximum tolerable doses of several chemotherapeutic agents, reaching a level of tumor toxicity far beyond any conventional consolidation usually administered [29–32]. Also, in one demonstrative Japanese study in particular, using a database of 2518 adult patients with AML in CR1, Kurosawa, Usuki et al. [33] conducted a 5-month landmark analysis after CR1 and compared 1290 patients who received chemotherapy alone with 103 who received ASCT . ASCT significantly improved 3-year RFS (58% vs. 37%; *P* < .001) but not OS.

Our study compared ASCT to haploidentical donors. It follows a previous EBMTstudy designed along the same lines, which compared in the same group of patients (intermediate-risk, CR1 MRD negative) ASCT and matched unrelated donors [24] and showed better OS for ASCT in patients with no FLT3-ITD. In the present paper we studied in the same risk and status group, whether in the absence of a matched sibling donor, one should use as alternative donors haploidentical donors rather than consider an ASCT: We report that in patients with no FLT3-ITD, indeed the results are in favor of ASCT with the same LFS, less NRM and a better OS. In addition ASCT requires no donor search, is technically easier, and is associated with less toxicity (no GVH) a lower NRM and a better cost/effectiveness ratio.

There has been in the past twenty years or so considerable development in our understanding of AML and considerable development in the field of stem cell transplantation, that must be taken into account when comparing different therapeutic strategies retrospectively and planning for the future:

First, regarding AML is has been recognized as a highly heterogeneous disease. In the past two decades, our understanding of the disease has considerably benefited from the development of cytogenetics, flow cytometry and molecular biology including next generation sequencing. Several mutational markers of prognostic or therapeutic significance such as NPM1, FLT3-ITD, IDH1 and IDH2, P53, overexpression of BCL2 as well as many others have been identified. Prognostic classifications have been built using all this information and regularly updated, leading to the individualization of three risk categories: favorable, intermediate and adverse [1, 34–38]. Also targeted therapies have been developed but the heterogeneity of the disease and the existence of several clones and subclones emerging continuously may be taken in favor of myeloablative therapies such as provided by ASCT or used in about 50% of present haploidentical donor transplants.

Second, the concept of CR has moved forward with the new goal being to reach a status of MRD negativity. Indeed, patients in MRD negative CR after induction, at time of transplant, or after transplant have been shown to be at lower risk of subsequent relapse [2–4].

Third, regarding allogeneic stem cell transplantation, there have been constant improvements: the shift from Bone Marrow to Peripheral Blood as a preferred source of stem cells, the development of reduced intensity conditioning leading to a decreased risk of NRM, and most of all the use of alternative sources of stem cells such as haploidentical donors have rendered allogeneic HSCT available to most patients up to 70 and even 75 years of age. Of importance, several retrospective and prospective studies comparing haploidentical donor HSCT to other sources of allogeneic stem cells including cord blood, unrelated donors and even matched sibling donors have shown similar or even better results with HAPLO in high-risk patients [39–45]. Presently, most guidelines recommend consolidation with allogeneic stem cell transplantation for all non-favorable-risk patients either in CR1 or after relapse [1, 27, 28]. There remains however, two matters of concern after haploidentical donor HSCT, which are a residual risk of relapse post-transplant of 10- 30% and a risk of severe chronic GVHD of around 10-20%, which reduces the GVHD-free and relapse-free survival (GRFS) and negatively impacts quality of life [42, 46]. Attempts at reducing the RI are presently testing the role of maintenance therapy post-transplant with various agents such as FLT3 inhibitors or targeting other existing molecular abnormalities [14–16] or new combinations such as the combination of 5-azacytidine with venetoclax [12] possibly combined to the anti-CD47 monoclonal antibody magrolimab [13].

Finally, ASCT has benefited from less improvement: it has been widely used to consolidate patients with AML in remission before the year 2000 [23, 47, 48], but although it has been shown to be associated with a much lower NRM than allogeneic HSCT, its major impediment has remained the higher rate of relapses post-transplant [49]. Importantly, historical randomized studies as well as many retrospective registry studies have shown a significant reduction in the RI postASCT over conventional chemotherapy [29–32] and its potential role in selected patient groups such as rapid remitters [50], patients with acute promyelocytic leukemia in CR2 [51], and favorable-risk patients [52–56] including those with double mutant CEBPA [57, 58], The potential role of ASCT in intermediate-risk patients and more widely in all patients in CR1 and MRD negative has been highlighted by many teams. Nonetheless, the very successful development of allogeneic transplantation, as described above, has resulted in a considerable decrease in the numbers of ASCT done each year and ASCT for AML has become somewhat out of fashion. Indeed, the EBMT registry collects annually no more than 300 ASCT for myeloid malignancies [59]. Importantly, some developments for ASCT have however occurred in the past decade which have included in particular, an improvement of the pretransplant regimen with intravenous busulfan combined with melphalan [60–62] or the BEA (Busulfan, Etoposide,ARA-C) regimen [63] and as mentioned above, a better definition of the AML patient population that may benefit from ASCT.

When comparing ASCT to allogeneic transplantation, there have been several retrospective studies of patients with intermediate-risk AML showing similar results with ASCT or allogeneic HSCT, in particular from Japan [64, 65], and the Hovon group [66, 67]. Two single center studies from China [68, 69] also reported a LFS of around 70% in patients with intermediate-risk AML autografted in CR1 with MRD negative.

Recently, the results of the GIMEMA 1013 trial were reported in which post-remission therapy of young patients with de novo AML was decided by combining cytogenetics and post-consolidation levels of MRD [70]. In this Italian trial, intermediate-risk patients received an ASCT if MRD negative or an allogeneic HSCT if MRD positive. The 2-year OS and LFS were 79% and 61% respectively, in the intermediate-risk MRD-negative category, and 70% and 67% respectively, in the MRD positive category. The investigators concluded that ASCT may have a role in favorable- and intermediate-risk MRD-negative patients. In these studies haploidentical transplants were not considered.

In the present study which compared ASCT to haploidentical donor HSCT, we showed that, in patients with no FLT3 mutation, transplant outcomes were similar for LFS, and the OS significantly better with ASCT; we also showed in contrast that, in patients with FLT3-ITD, haploidentical donor HSCT had better outcome. The present study follows a similar previous EBMT registry study in patients in first molecular remission, comparing at that time the outcome postASCT and the outcome post-allotransplant with matched unrelated donors [24]. This previous study had reached similar conclusions using the ELN 2017 risk classification, showing similar LFS in the absence of FLT3-ITD (favorable and intermediate-risk category 2) and better OS with ASCT in patients in the favorable-risk group, but better results with unrelated matched donors in patients with FLT3-ITD (intermediate-risk category 1).

FLT3-ITD is one of the first identified molecular marker initially associated with poor prognosis [71] and this was clearly verified in our study. Not surprisingly, transplant teams tended to favor haploidentical donor HSCT for FLT3-ITD patients, and this potentially has contributed to the interaction between FLT3 mutation status and outcomes.

The stratified comparisons in our study indeed showed differences in outcomes between FLT3-wt and FLT3-ITD patients, supporting the use of a FLT3-adapted therapeutic strategy.

Recently, there has been considerable evidence demonstrating that FLT3-ITD patients can benefit from inhibitors at induction and/or for maintenance post-HSCT [14–22] In fact, according to the recently updated European Leukemia Net recommendations for adult AML, the FLT3-ITD allelic ratio is no longer considered in the risk classification and FLT3-ITD is now categorized as an intermediate-risk marker, irrespective of the allelic ratio or the concurrent presence of NPM1 mutation, because of methodological issues in detection, the growing use of FLT3 inhibitors, and therapeutic decisions increasingly directed by MRD level. Hence, even for patients with FLT3-ITD who achieve MRD negativity, whether ASCT combined with an FLT3 inhibitor may become a valid alternative option to allogeneic HSCT remains to be studied.

This study has the usual limitations of all retrospective registry studies.

To these may be added the lack of details on MRD assessment and the absence of evaluation of FLT3 inhibitors. Regarding MRD, the MRD detection methods used were heterogeneous; despite this heterogeneity which was confirmed in a recent EBMT survey [72], the huge impact of MRD as reported to the registry on the outcome, has been similarly found previously in several other recent EBMT studies [73–75]. Regarding the absence of data concerning the use of FLT3 inhibitors, it does not concern the FLT3-wt group but it may be of importance in the FLT3-ITD group for which haploidentical donor transplants are associated with a better outcome. Whether the use of FLT3 inhibitors might further improve the outcome post haploidentical donor transplants, or postASCT or both and comparative studies will be of great interest but feasible only when we get sufficient data in particular in the ASCT arm. Unfortunately, we do not presently have enough data and follow-up in the registry to address this additional important question.

The present study was not designed to compare ASCT to conventional chemotherapy. A new “modern” prospective randomized study with the “tools of today” would probably be of great interest.

We conclude from the present study that adult patients with AML in the intermediate-risk category with no FLT3-ITD and in CR with no detectable MRD can be offered ASCT as a therapeutic option. Recent results of maintenance therapy with novel drugs post-allogeneic HSCT [14, 22, 76, 77] suggest a similar approach might be appropriate postASCT to reduce the RI.

The contribution of maintenance therapy postASCT and post haploidentical donors deserves further investigation by well-designed prospective studies.

### Supplementary information

Methods used to classify 393 patients in first remission as negative for the detection of minimal residual disease (MRD negative)
