# Supplementary material for: Autologous stem cell transplantation in adult patients with intermediate-risk acute myeloid leukemia in first complete remission and no detectable minimal residual disease. A comparative retrospective study with haploidentical transplants of the global committee and the ALWP of the EBMT
Source: Bone Marrow Transplant. 2023 Aug 28;58(12):1322–30. doi: 10.1038/s41409-023-02070-9 (PMC10691968; doi:10.1038/s41409-023-02070-9)
Supplement: Supplementary file 1 — Methods used to classify 393 patients in first remission as negative for the detection of minimal residual disease (MRD negative) [file 41409_2023_2070_MOESM1_ESM.docx]

**Supplementary table 1**

**METHODS USED TO CLASSIFY 393 PATIENTS IN FIRST REMISSION AS NEGATIVE FOR THE DETECTION OF MINIMAL RESIDUAL DISEASE (MRD negative)**

A: Repartition in the two groups: autologous and haploidentical transplants

| Method | **Haplo** | **Auto** | ***Total*** |
| --- | --- | --- | --- |
| PCR +-/ NGS | 27 (16.4%) | 19 (8.3%) | ***46*** |
| FACS only | 20 (12.1%) | 46 (20.2%) | ***66*** |
| PCR+FACS | 67 (40.6%) | 75 (32.9%) | ***142*** |
| PCR+FACS+NGS | 51 (30.9%) | 88 (38.6%) | ***139*** |
| ***Total*** | ***165*** | ***228*** | ***393*** |

B: Repartition in the two groups: FlT3-wt and FLT3-ITD

| Method | **FLT3-wt** | **FLT3-ITD** | ***Total*** |
| --- | --- | --- | --- |
| PCR +-/ NGS | 31 (12.4%) | 15 (10.4%) | ***46*** |
| FACS only | 42 (16.9%) | 24 (16.7%) | ***66*** |
| PCR+FACS | 91 (36.5%) | 51 (35.4%) | ***142*** |
| PCR+FACS+NGS | 85 (34.10%) | 54 (37.5%) | ***139*** |
| ***Total*** | ***249*** | ***144*** | ***393*** |

PCR: Polymerase chain reaction; FACS: Fluorescence activated cell sorter; NGS: Next generation, sequencing.
